# Robust Real-Time Sperm Tracking with Identity Reassignment Using Extended Kalman Filtering

**DOI:** 10.3390/s25247539

**Published:** 2025-12-11

**Authors:** Mahdieh Gol Hassani, Mozafar Saadat, Peiran Lei

**Affiliations:** Department of Mechanical Engineering, School of Engineering, University of Birmingham, Birmingham B15 2TT, UK; m.saadat@bham.ac.uk (M.S.); pxl165@student.bham.ac.uk (P.L.)

**Keywords:** multi-object tracking, Extended Kalman Filter, identity reassignment, microscopy video analysis, sperm tracking, computer vision in assisted reproductive technology

## Abstract

Accurate and real-time sperm tracking is essential for automation in Intracytoplasmic Sperm Injection (ICSI) and fertility diagnostics, where maintaining correct identities across frames improves the reliability of sperm selection. However, identity fragmentation, overcounting, and tracking instability remain persistent challenges in crowded and low-contrast microscopy conditions. This study presents a robust two-layer tracking framework that integrates BoT-SORT with an Extended Kalman Filter (EKF) to enhance identity continuity. The EKF models sperm trajectories using a nonlinear state that includes position, velocity, and heading, allowing it to predict motion across occlusions and correct fragmented or duplicate IDs. We evaluated the framework on microscopy videos from the VISEM dataset using standard multi-object tracking (MOT) metrics and trajectory statistics. Compared to BoT-SORT, the proposed EKF-BoT-SORT achieved notable improvements: IDF1 increased from 80.30% to 84.84%, ID switches reduced from 176 to 132, average track duration extended from 74.4 to 91.3 frames, and ID overcount decreased from 68.75% to 37.5%. These results confirm that the EKF layer significantly improves identity preservation without compromising real-time feasibility. The method may offer a practical foundation for integrating computer vision into ICSI workflows and sperm motility analysis systems.

## 1. Introduction

Infertility is a condition affecting the reproductive system, covering a broad range of issues that impair an individual’s ability to conceive. This condition approximately affects 12–28% of couples within the reproductive age group worldwide. Male infertility is responsible for about 40–50% of the documented cases, both as the main or as a contributing cause, affecting at least 7% of men [1,2]. Sperm motility is a key parameter in assessing male fertility and plays a crucial role in Assisted Reproductive Technologies (ART), including In Vitro Fertilization (IVF), Artificial Insemination (AI), and Intracytoplasmic Sperm Injection (ICSI) [3]. The ability of spermatozoa to navigate the environment of the female reproductive tract directly influences fertilization success [4]. Reliable sperm tracking enables quantitative evaluation of motility parameters and is essential not only for clinical diagnostics but also for advancing our fundamental understanding of sperm biomechanics and function [5,6,7].

Among ART procedures, Intracytoplasmic Sperm Injection (ICSI) is one of the most widely used treatments for male-factor infertility. In ICSI, a single sperm is selected and immobilized by scraping its tail using a micropipette, then aspirated into the micropipette to be injected directly into a mature oocyte. The micropipette penetrates the zona pellucida and punctures the oolemma to deposit the sperm into the cytoplasm, aiding fertilization [8]. Although ICSI can achieve success rates of up to ~37% per embryo transfer, outcomes are highly dependent on the embryologist’s skill during critical steps such as sperm selection, immobilization, and precise injection [8,9]. Human error or operator variability in these stages can lead to fertilization failure, oocyte degeneration, or reduced embryo viability [9,10,11,12].

Automating parts of the ICSI workflow, particularly sperm selection, has the potential to reduce operator dependency, improve consistency, and increase treatment success rates [8,13,14]. A robust, real-time sperm tracking system capable of maintaining identity under dense, nonlinear, and low-contrast imaging conditions is a core requirement for such automation. This capability not only supports motility-based sperm selection for ICSI but also improves the reliability of downstream Computer-Aided Sperm Analysis (CASA) measurements.

Conventional Computer-Aided Sperm Analysis (CASA) systems automate sperm trajectory extraction by detecting and linking sperm frame-by-frame. While effective in controlled settings, many CASA implementations struggle to maintain consistent sperm identities over time. High sperm density, frequent overlaps, and nonlinear swimming motions often cause identity switches and fragmented tracks, particularly in longer videos or more complex samples. These tracking errors limit the accuracy and reliability of motility assessments. Therefore, there is a need for advanced tracking methods that integrate robust detection with motion models capable of preserving identities under challenging conditions [15,16,17,18,19].

Sperm tracking is further complicated by the unique characteristics of sperm motion. Cells move quickly, often in dense populations, and appear with low contrast in noisy microscopy videos [5,20]. Overlaps and occlusions lead to repeated ID switches in traditional pipelines [15,21], while the nonlinear and nonrigid nature of swimming, including frequent heading changes and flagellar motion, adds additional complexity [4,22,23].

Recent advances in computer vision have contributed to progress in addressing these challenges. Deep learning-based object detectors, such as You Only Look Once (YOLO) [24], have achieved state-of-the-art accuracy in sperm detection, outperforming classical methods under variable imaging conditions [25,26]. On the tracking side, advanced multi-object tracking algorithms such as ByteTrack [27], BoT-SORT [28], and DeepSORT [29] leverage appearance, motion, and spatial-temporal cues to improve identity preservation and reduce switches more reliably in complex scenes [15,18,19]. Additionally, end-to-end transformer-based and graph-attention-based models leverage global feature reasoning and attention-based association to more effectively model object relationships across space and time. Transformer-based models, such as Transtrack detection and tracking, are unified onto one framework, performing joint learning [30]. In graph attention models such as TransMOT, objects represent nodes in spatial-temporal graphs; the interaction between objects is learned using edge relations [31]. Methods like DanceTrack have shown promise in tracking dense populations of visually similar objects with diverse motion patterns [32], a scenario similar to sperm tracking; however, it is designed for human-scale objects in RGB video and does not explicitly address the nonlinear rotational dynamics or low-contrast conditions of sperm microscopy videos.

These methods can show stronger accuracy on public (MOT) benchmarks by using global feature reasoning and attention-based association. However, these architectures typically have high computational cost and inference latency, which limits their application in real-time environments [30,31,33,34].

Extended Kalman Filters (EKFs) combine nonlinear motion models with noisy measurements to produce robust state estimates and have proven effective in tracking complex trajectories. It has been widely applied in fields such as robotics, biomedical imaging, pedestrian tracking, and autonomous driving for nonlinear state estimation and sensor fusion [16,35,36]. In typical 2D computer vision tasks, however, EKFs are less commonly employed due to the lack of orientation data and the dominance of deep learning–based trackers. In sperm tracking, EKF-based approaches remain unexplored; most methods still rely on linear displacement models without incorporating heading direction or rotational dynamics. This gap motivates our application of EKF to explicitly model nonlinear sperm swimming behaviour and maintain identity continuity in challenging microscopy videos.

The importance of interpretability and robustness of AI systems for the purpose of supporting clinical decisions has been highlighted in recent advancements of biomedical research. Adnan et al. [37] developed an explainable deep learning framework for brain tumour detection, by visually pointing to and quantifying predictions using LIME and Grad-CAM. Nasir et al. [38] proposed a Spiking Attention Block in skin cancer detection to integrate spatial and channel attention mechanisms that would improve reliability and explainability into convolutional neural networks; Shaik et al. [39] proposed a hybrid explainability framework by combining Shapley-value–based feature attribution with attention mechanisms to enhance interpretability in deep learning models for clinical decision support.

The aim of this study is to develop a robust sperm tracking approach capable of preserving identities under nonlinear motion, occlusion, and low contrast. We propose a two-layer framework that integrates BoT-SORT with an EKF-based verification module. BoT-SORT provides a strong baseline detection-to-track association, while the EKF refines trajectory predictions by explicitly modelling sperm heading direction and nonlinear motion. This layered approach improves identity preservation, reduces ID switches, and prevents overcounting, thereby supporting more informed fertility treatment decisions and potentially a better understanding of sperm biomechanics. We evaluate our method on the publicly available VISEM dataset, this dataset consists of video recordings of sperm performed by putting a sample on a heated microscope stage (37 °C). The sample was examined under a 400 times magnification with an Olympus CX31 microscope (Olympus Corporation, Hachioji, Tokyo, Japan). The videos were captured by a microscope-mounted camera (specifically a UEye UI-2210C made by IDS Imaging Development Systems, Obersulm, Germany) and saved as an AVI file [5].

The evaluation demonstrates improved tracking accuracy compared to standalone multi-object trackers. These improvements have the potential to enhance the reliability of CASA-derived motility parameters, ultimately supporting more accurate clinical diagnostics and advancing research into sperm biomechanics.

## 2. Materials and Methods

**Figure 1:**
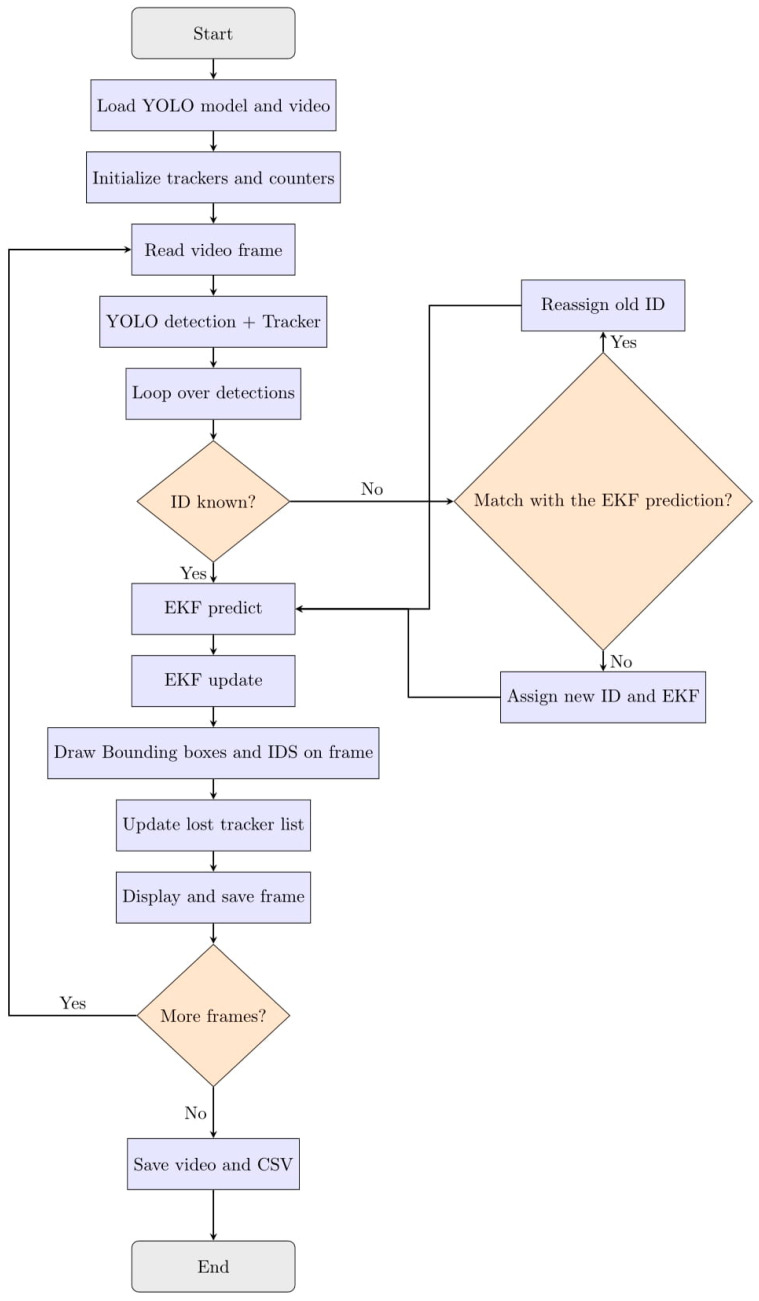
The overview of the EKF-BoT-SORT algorithm.

This work presents a robust two-layer sperm tracking framework that combines state-of-the-art object detection and multi-object tracking with an Extended Kalman Filter (EKF) verification layer. The pipeline is designed to minimize identity switches and overcounting, thereby providing reliable and biologically meaningful sperm trajectories for downstream motility analysis [20,36,40]. Figure 1 illustrates the pipeline flow, which consists of three main stages:

Object Detection: Each video frame is processed using YOLO11m, a fast and accurate deep learning–based detector optimized for microscopy images of sperm cells [41,42,43]. YOLO11 outputs bounding boxes and confidence scores for detected sperms.

Object Tracking: Initial object identity assignment and frame-to-frame tracking are performed using baseline multi-object tracking algorithms, primarily BoT-SORT. BoT-SORT is evaluated both in its standard form and integrated with the EKF verification layer. These trackers are chosen due to their complementary strengths and proven robustness in crowded and occluded scenes [15]. BoT-SORT extends the classical SORT framework by incorporating camera motion compensation and a two-stage association process, making it well-suited to the dense and dynamic sperm tracking environment [15,44,45]. For comprehensive benchmarking, additional trackers such as ByteTrack and DeepSlORT are also included in the evaluation [27,29].

EKF-Based Long-Term Verification: Running in parallel with short-term tracking, the EKF module predicts sperm trajectories by modelling nonlinear motion dynamics, including position, velocity, and heading direction. When an object is temporarily lost and subsequently detected again with a new ID, the EKF compares the predicted state with the new detection. If suitable, the original ID is reassigned, correcting identity fragmentation and preventing overcounting [36,40].

To investigate the different types of tracking systems and whether or not they are suitable for performing real-time sperm tracking in clinical environments, we performed a qualitative assessment on representative tracker families, highlighting their core mechanisms, strengths, and limitations in relation to real-time sperm tracking performance.

Table 1 provides an overview of classical, transformer-based, and graph-based trackers, their key mechanisms, real-time performance, and overall practical suitability. While the methods are used in different applications, the table provides a general overview of the methods’ performance. The HDE-Track [34] method has been modified specifically for sperm tracking and has provided a quantitative comparison of how different transformer-based and Kalman filter-based models perform in sperm tracking.

As shown in Table 1, classical Kalman Filter–based methods (BoT-SORT, ByteTrack, EKF-BoT-SORT) maintain real-time operation, making them practical for applying online tracking on dense sperm videos. In contrast, end-to-end transformer and graph-attention trackers operate below real-time thresholds (<15 FPS) but with better accuracy, suitable for offline analysis. In this study, we selected classical multi-object trackers such as BoT-SORT, ByteTrack, and DeepSORT for comparative evaluation, as they are robust, more computationally efficient for real-time tracking, and fall into the classical Kalman-based tracking methods [27,29,30,31,33,34].

### 2.1. YOLO11 Detection

To perform frame-wise detection of spermatozoa, we use YOLO11, a real-time object detection model that offers an effective balance between speed and accuracy, making it well-suited for tracking small, fast-moving objects in microscopy videos [41,42]. YOLO11 was trained on annotated videos from the VISEM dataset [5], using 300 epochs with early stopping based on validation loss to prevent overfitting. The standard built-in Non-Maximum Suppression (NMS) step in YOLO11 suppressed the duplicate detections [24]. The checkpoint achieving the best validation performance was selected for integration into the tracking pipeline. YOLO11’s high recall ensures that the majority of the sperm are consistently detected across frames, which is critical for preserving long-term identity in the tracking stage. Its lightweight design allows for high-throughput, real-time analysis, making it suitable for practical use in automated sperm assessment tools [5,7].

### 2.2. BoT-SORT Baseline Tracker

BoT-SORT (Bag of Tricks for SORT) is a robust multi-object tracking algorithm that builds upon the foundational Kalman Filter-based tracking framework SORT, with several enhancements inspired by DeepSORT and ByteTrack. These modifications improve prediction accuracy, handle camera motion, and leverage multiple association strategies to ensure more reliable tracking [28]. BoT-SORT incorporates camera motion compensation to distinguish object movement from background changes and refines the Kalman Filter state vector for improved modelling of object dynamics [46]. Such improvements make it particularly effective in crowded and dynamic environments, including dense sperm tracking scenarios [47,48]. Its robust data association framework reduces identity switches by efficiently matching detections across frames, even under occlusion or low-confidence conditions [28,47], and has demonstrated strong performance on multi-object tracking benchmarks and in medical imaging applications [49].

#### 2.2.1. BoT-SORT Input and Detection Filtering

**Figure 2:**
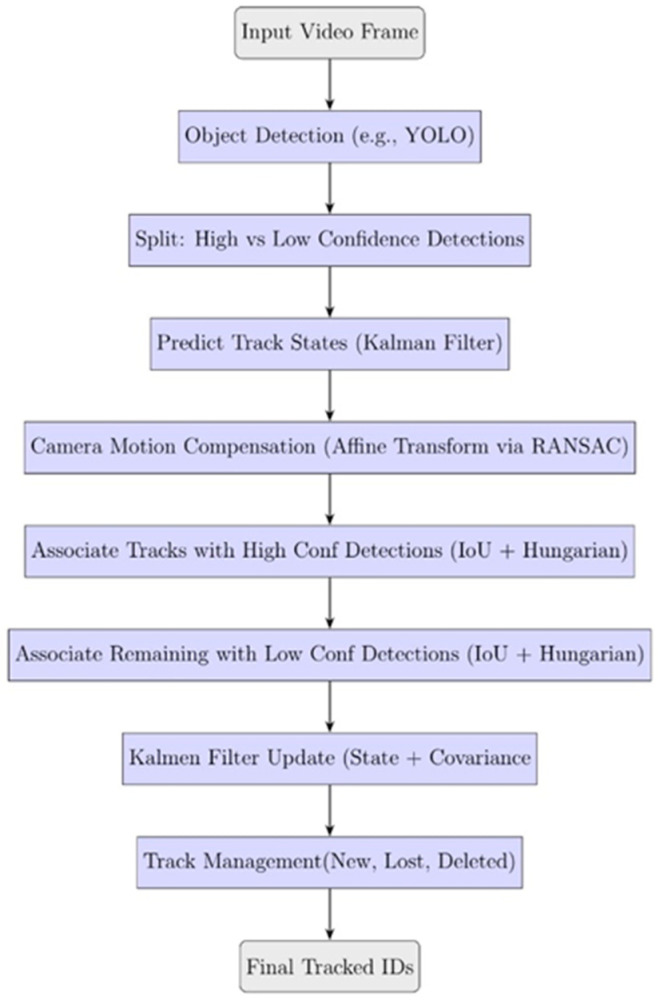
Overview of the BoT-SORT algorithm.

BoT-SORT operates under the widely adopted tracking-by-detection paradigm, in which detections are provided for each frame as bounding boxes with associated confidence scores, typically from a deep learning–based detector such as YOLO11 [45,46]. These detections are split into two categories based on confidence levels: high-confidence detections are associated with active tracks using Intersection-over-Union (IoU) as the cost metric. The Hungarian algorithm is applied to solve the assignment problem optimally, with matches having an IoU below a predefined threshold τ being discarded to avoid incorrect associations. These detections are used in the primary data association stage, while in the second stage (Secondary Matching), unmatched tracks from the first stage are associated with low-confidence detections falling between η  and τ, again using IoU and the Hungarian algorithm. This two-stage association allows BoT-SORT to recover missed detections and reduce false negatives, which is particularly important in crowded or partially occluded settings [28,45,46,49,50]. Track lifecycle management follows a stable strategy to minimize identity switches and premature deletion. Tracks are initialized from unmatched high-confidence detections and promoted to “confirmed” status only after multiple consecutive matches. Unmatched tracks are marked as “lost” after a fixed number of missed frames and are deleted if unmatched for a longer period. Occasional duplicate detections remaining after YOLO11’s NMS are automatically resolved during BoT-SORT’s association step, where detections referring to the same object are suppressed through motion-consistency checks and track-management filtering. Duplicate tracks are eliminated based on spatial overlap [6,28]. Figure 2 illustrates the BoT-SORT tracking pipeline.

#### 2.2.2. BoT-SORT State Representation and Prediction

Each tracked object is modelled using an 8-dimensional state vector:

(1) $$x={\left[\begin{array}{cccccccc} x_{c} & y_{c} & w & h & {\dot{x}}_{c} & {\dot{y}}_{c} & \dot{w} & \dot{h} \end{array}\right]}^{T}$$

where $\left(x_{c},y_{c}\right)$ are the bounding box centre coordinates, w and h represent its width and height, and the remaining components correspond to their respective velocities [46,51]. Motion prediction is predicted using a Kalman Filter under a constant velocity assumption:

(2) $${\hat{x}}_{k|k-1}=Fx_{k-1},P_{k|k-1}=FP_{k-1}F^{T}+Q$$

where  P is the state covariance matrix, and Q represents the process noise covariance, capturing uncertainty in the motion model [51,52]. This prediction step allows the tracker to estimate future locations even with missed detections, and the recursive Kalman Filter update refines estimates when new measurements arrive [45,46].

#### 2.2.3. Camera Motion Compensation

To handle jitter or panning in moving-camera setups, BoT-SORT includes an optional CMC module. This module estimates a global affine transformation between consecutive frames by detecting and matching background keypoints, then applies RANSAC to remove outliers. The inverse transformation is applied to all predicted track positions, ensuring that data association considers only object motion relative to the scene [28,45,46].

#### 2.2.4. Kalman Filter Update

Matched detections are used to correct the predicted states using the Kalman Filter update equations. The loss (difference between detection and prediction) is computed and used to update both the state and its uncertainty:

(3) $$y_{k}=z_{k}-H{\hat{x}}_{k|k-1}$$

(4) $$K_{k}=P_{k|k-1}H^{T}{\left(HP_{k|k-1}H^{T}+R\right)}^{-1}$$

(5) $${\hat{x}}_{k|k}={\hat{x}}_{k|k-1}+K_{k}y_{k}$$

(6) $$P_{k|k}=(I-K_{k}H)P_{k|k-1}$$

where H is the observation matrix, $K_{k}$ is the Kalman gain, and R is the measurement noise covariance [50,53].

#### 2.2.5. BoT-SORT Configuration

To define the suitable values for high and low-confidence thresholds (*τ*, *η*) of BoT-SORT’s two-stage association, a sensitivity analysis has been conducted. These parameter settings determine how the detections from YOLO11 are classified as reliable or uncertain before track association is performed. The analysis was performed on videos with different sperm densities, varying from low to medium and high densities [28].

The high-confidence threshold was varied between *τ* ∈ [0.25, 0.70] and the low confidence threshold varied between *η* ∈ [0.05, 0.20], consisting of both strict and more lenient detection settings, while keeping all the other parameters fixed [27,28]. Tracking performance was evaluated using the standard MOT metrics (IDF1, ID switches, MOTA, and MOTP) to show the influence of *τ* and *η* on identity preservation and tracking accuracy.

As shown in Appendix A, Table A1, varying *τ* and *η* within the tested ranges has only a small impact on tracking accuracy. Increasing *τ* from 0.25 to 0.60 improved precision and reduced identity switches, while high values decreased recall. Table A2 in Appendix A shows that changes in *η* between 0.05 and 0.20 make small differences (<3%) in IDF1 and MOTA.

The configuration *τ* = 0.50 and *η* = 0.10 achieved the best balance between recall and identity stability across all sperm densities. This setting was adopted as the default configuration for all experiments.

### 2.3. State Estimation Using the Extended Kalman Filter (EKF)

To obtain accurate and temporally consistent position and velocity estimates for individual sperm cells, we employed an Extended Kalman Filter (EKF) as a recursive state estimator. While object detection models such as YOLO provide instantaneous (x,y) positions, these measurements are subject to noise and lack temporal continuity. The EKF addresses this by fusing current detections with motion-based predictions from previous frames, resulting in a smoother and more reliable trajectory [45,46,54,55]. This fusion is especially valuable in noisy microscopy videos, where detection fluctuations can lead to unstable tracks. EKF’s ability to filter measurement noise and maintain consistent object motion has been demonstrated in other dynamic tracking domains [56,57], making it well-suited for our application.

#### 2.3.1. State Representation

The state vector $x_{k}\in R^{5}$ at time step  k is defined as

(7) $$x_{k}={\left[\begin{array}{ccc} x & y & v_{x} \end{array}\;\;\;\begin{array}{cc} v_{y} & \theta \end{array}\right]}^{T}$$

where (x,y) represents the 2D position of a sperm cell, $(v_{x},v_{y})\;$ are the velocity components, and θ captures the orientation. The motion model assumes constant velocity, and the nonlinear transition function f(x) is given by:

(8) $$f(x)=\left[\begin{array}{c} x+\Delta t.v_{x}\\ y+\Delta t.v_{y}\\ \begin{array}{c} v_{x}\\ \begin{array}{c} v_{y}\\ \theta \end{array} \end{array} \end{array}\right]$$

Here, Δt is the time interval between frames. This simplified model provides a computationally efficient yet sufficiently accurate approximation of sperm motion, especially when paired with process noise to account for deviations from idealized motion.

The Jacobian of the motion model $F_{k}=\frac{\partial f}{\partial x}$ is:

(9) $$f(x)=\left[\begin{array}{ccccc} 1 & 0 & \Delta t & 0 & 0\\ 0 & 1 & 0 & \Delta t & 0\\ 0 & 0 & 1 & 0 & 0\\ 0 & 0 & 0 & 1 & 0\\ 0 & 0 & 0 & 0 & 1 \end{array}\right]$$

Such state representations and motion models are commonly used in Extended Kalman Filter frameworks for applications in biomedical imaging, robotics, and navigation systems [36,54,56].

#### 2.3.2. Measurement Model

The observations are 2D position measurements $z_{k}={\left[\begin{array}{cc} x & y \end{array}\right]}^{T}$, obtained from the detection pipeline (e.g., YOLO). The measurement function h(x) is:

(10) $$h(x)=\left[\begin{array}{c} x\\ y \end{array}\right]$$

Its Jacobian $H_{k}=\frac{\partial h}{\partial x}$ is:

(11) $$H(x)=\left[\begin{array}{c} \begin{array}{ccccc} 1 & 0 & 0 & 0 & 0 \end{array}\\ \begin{array}{ccccc} 0 & 1 & 0 & 0 & 0 \end{array} \end{array}\right]$$

This linear measurement model assumes that only position components are directly observed, while velocity and orientation are estimated indirectly, a common approach in Kalman-based trackers [36,54,56].

#### 2.3.3. Noise Covariance Matrices

The process noise covariance $Q_{k}$ and measurement noise covariance $R_{k}$ are empirically tuned to balance responsiveness with trajectory smoothness:

(12) $$Q_{k}=\sigma_{q}^{2}\cdot I_{4\times 4\;},\;\;R_{k}=\sigma_{r}^{2}\cdot I_{4\times 4\;}$$

where $\sigma_{q}$ and $\sigma_{r}$ represent assumed standard deviations of process and measurement noise, respectively [36,56,58].

#### 2.3.4. Recursive Update

The EKF follows the standard prediction-update cycle [36,54,56]:

Prediction:

(13) $${\hat{x}}_{k}^{-}=f\left({\hat{x}}_{k-1}\right)$$

(14) $$P_{k}^{-}=F_{k}P_{k-1}F_{k}^{T}+Q_{k}$$

(15) $${\hat{x}}_{k|k-1}=Fx_{k-1}$$

(16) $$P_{k|k-1}=FP_{k-1}F^{T}+Q$$

Update:

(17) $$K_{k}={P_{k}^{-}H_{k}^{T}\left(H_{k}P_{k}^{-}H_{k}^{T}+R_{k}\right)}^{-1}$$

(18) $${\hat{x}}_{k}={\hat{x}}_{k}^{-}+K_{k}(z_{k}-h({\hat{x}}_{k}^{-}))$$

(19) $$P_{k}=(I-K_{k}H_{k})P_{k}^{-}$$

This framework allows for robust estimation of position and velocity, even when detection quality fluctuates or two sperms pass in close proximity.

Algorithm 1 summarizes the EKF implementation used for 2D sperm tracking with position and heading estimation. This implementation follows a standard predict-update cycle incorporating heading updates and nonlinear motion modelling tailored to the swimming dynamics of sperm. The framework constitutes the proposed tracking method in this work.
**Algorithm 1** EKF algorithm for 2D Position and Heading**Require:** Initial state ${\dot{x}}_{0}={\left[x,y,v,\theta \right]}^{T}$, covariance $P_{0}$, Process noise Q, measurement noise R1: **Function** Predict2: Predict State: ${\dot{x}}_{k}^{-}=\;\left[\begin{array}{c} x_{k-1}+v_{k-1}∆t\cos\theta_{k-1}\\ y_{k-1}+v_{k-1}∆t\cos\theta_{k-1}\\ \begin{array}{c} v_{k-1}\\ \theta_{k-1} \end{array} \end{array}\right]$
3: Compute Jacobian: $F_{k}=\left[\begin{array}{cccc} 1 & 0 & ∆t\cos\theta & -v∆t\sin\theta \\ 0 & 1 & ∆t\sin\theta & -v∆t\cos\theta \\ 0 & 0 & 1 & 0\\ 0 & 0 & 0 & 1 \end{array}\right]$
4: Predict covariance: $P_{k}^{-}=F_{k}P_{K-1}F_{k}^{T}+Q$
5: **end function**6: **function** Update ($z_{k}$)7:   Measurement Model: $h\left({\dot{x}}_{k}^{-}\right)=\;\left[\begin{array}{c} x\\ y \end{array}\right]$
8:  Measurement Jacobian: $H_{k}=\;\left[\begin{array}{cccc} 1 & 0 & 0 & 0\\ 0 & 1 & 0 & 0 \end{array}\right]$
9:  Kalman gain: $K_{k}=\;P_{k}^{-}H_{k}^{T}{\left(H_{k}P_{k}^{-}H_{k}^{T}+R\right)}^{-1}$
10:  State update: ${\dot{x}}_{k}=\;{\dot{x}}_{k}^{-}+K_{k}(z_{k}-h\left({\dot{x}}_{k}^{-}\right))$
11:  Covariance update: $P_{k}=(I-K_{k}H_{k})P_{k}^{-}$
12:
  **If** 
$\left\|\left[\begin{array}{c} v\cos\theta \\ v\sin\theta \end{array}\right]\right\|>10^{-4}$
 **then**
13:    
$\theta \;\leftarrow \;\tan^{-1}\;2(v\sin\theta ,v\cos\theta )$
14
  **end if**
15: **end function**16: **function** Velocity17:  
**return** 
$\dot{v}=\left[\begin{array}{c} v\;\cos\theta \\ v\sin\theta \end{array}\right]$
18: **end function**19: **function** Loss (**z**)20:  
$\dot{z}\leftarrow \;\left[\begin{array}{c} x\\ y \end{array}\right]$
21:  
**return** 
${\left\|z-\dot{z}\right\|}^{2}$
22: **end function**

In this framework, the state transition follows a nonlinear kinematic model:

(20) xt+1=xt+vtcos(θt)Δt,yt+1=yt+vtsin(θt)Δt,vt+1=vt,θt+1=θt

In this formulation, both position and velocity are functions of the current orientation, which allows the curvilinear and rotational swimming behaviour of sperm motion to be captured. This orientation-dependent model allows the predicted trajectory to follow the swimming path, including the circular or helical movement patterns of sperm, which is unlike the constant-velocity (linear) model that assumes movement along the fixed Cartesian axes.

The nonlinear transition is linearized at each time step using the Jacobian of the motion model, which allows the EKF to estimate the uncertainty analytically. The small variations in velocity and orientation, which have not been modelled, are accounted for using the process noise covariance Q, allowing the filter to adapt to small deviations in motion while maintaining stability. This formulation aligns the predicted motion with realistic sperm movement rather than a simple linear motion assumption, which improves trajectory continuity and ID consistency.

To evaluate the stability of the Extended Kalman Filter, a sensitivity analysis was conducted on the process noise covariance Q and measurement noise covariance R. Both parameters were varied across the scales of {0.5, 1.0, 2.0}, while all the other parameters were fixed. The range of {0.5, 1.0, 2.0} shows configurations for both 50% lower and 100% higher noise levels assumed compared to the baseline settings. For each of the Q and R configurations, the analysis was performed on videos with low, medium, and high-density levels, and the standard MOT metrics were computed, allowing the effect of changes in noise levels on identity preservation and tracking performance to be quantified [57].

The results of this analysis (see Appendix B, Table A3) show that changes in the noise covariances have minimal impact on tracking accuracy. Across all densities, the IDF1 and MOTA variations have stayed below 3%, which shows that the EKF’s stability with small to moderate changes in the noise assumptions. The configuration of α=1.0  and β=1.0 consistently provided the highest stability and balanced response; therefore, it was selected as the final setting for all following experiments. This shows that the EKF generalizes well across different sperm densities.

### 2.4. EKF Identity Reassignment

In sperm tracking systems, maintaining consistent identities across frames is critical for accurate motility analysis. However, standard detection and tracking approaches (e.g., YOLOv11 + BoT-SORT) often suffer from identity switches and overcounting, particularly when detections are temporarily lost due to occlusion or motion blur, or when two similar objects pass by each other, assigning a new ID to these objects [27,50]. In the context of sperm motility analysis, this leads to artificial inflation of the total sperm count, fragmented trajectories, and unreliable tracking data for any downstream analysis [34,51]. To address these issues, we introduce a two-layer verification where EKF operates on top of the BoT-SORT tracking to preserve identities.

Each track maintains an EKF-based prediction model. When a detection is lost or new detection appears with an unrecognized BoT-SORT ID, the Euclidean distance between its position $z_{k}^{\left(j\right)}$ and each lost track’s predicted state ${\hat{x}}_{k|k-1}^{\left(i\right)}$ is computed:

(21) $$If∥{\hat{x}}_{k|k-1}^{\left(i\right)}-z_{k}^{\left(j\right)}∥<\tau \Rightarrow {ID}_{j}\leftarrow {ID}_{i}$$

Here, τ is a dynamic threshold derived from the EKF’s uncertainty $P_{k|k-1}^{\left(i\right)}$ [36,59]. The predicted state represents the EKF’s estimate of the sperm’s next position, while the observed state corresponds to the centroid coordinates detected by YOLO in the current frame. The EKF combines both to correct prediction errors. If a match is found, the new detection is reassigned to the original ID, and the temporary BoT-SORT ID is discarded. To ensure data integrity, only EKF-validated IDs are retained for final analysis and display. This approach ensures:

- ID Persistence: Reduces identity switches caused by occlusions or brief detection gaps.
- Count Accuracy: Prevents the same sperm from being counted multiple times.
- Trajectory Coherence: Maintains uninterrupted motion paths, essential for any downstream analysis.

Temporary IDs introduced by BoT-SORT during detection dropouts are suppressed, avoiding overcounting.

To evaluate the stability of the reassignment threshold τ, we varied its value of τ across {15, 30, 45} pixels and evaluated IDF1, IDSW, and MOTA values over videos representing low, medium, and high sperm densities. The results are summarized in Table 2.

The variation in the threshold within the tested range shows minimal changes in tracking accuracy. IDF1 and MOTA show less than 3% variation across all densities, confirming the stability of the reassignment mechanism. The best balance between accurate ID recovery and minimal false reassignments was achieved by *τ* = 30 px and was therefore used as the default configuration for the following experiments.

The detailed steps of the proposed tracking framework are outlined in Algorithm 2, which presents the EKF-based ID reassignment logic.
**Algorithm 2** YOLO-Based Multi-Object Tracking with EKF and ID Reassignment**Require:** Pre-trained YOLO model, video stream, loss threshold δ, mas lost frame buffer L1: Initialize: frame counter k ←0, ID counter i←0
2: Initialize: EKF trackers T ← ∅, lost trackers  L ← ∅, ID mapping M←∅
3: **While** video frame available **do**4:     
k←k+1
5:    Read frame $I_{k}$ from video stream6:    Run YOLO +BoT-SORT to get detections: ${\left\{\left(b_{j},{id}_{j},c_{j}\right)\right\}}_{j=1}^{N_{k}}$
7:    **for** each detection j **do**8:      Compute centroid $z_{j}\leftarrow centre\;of\;b_{j}$
9:    
**if** 
${id}_{j}\notin M\;then$
10:      
best_match ←None,min_loss ←∞ 
11:      **for** each lost ID l∈L **do**12:          
${\hat{z}}_{l}\leftarrow {EKF}_{l}.predict()$
13:          
${loss}_{l}\leftarrow \;\left\|z_{j}-{\hat{z}}_{l}\right\|$
14:          
**If** 
${loss}_{l}<\delta \;and\;{loss}_{l}<min\text{\_}loss\;$
**then**
15:             
$best\text{\_}match,\;min\text{\_}loss\;\leftarrow {loss}_{l}$
16:          
**end if**
17:      
**end for**
18:      
**if** 
best_match ≠none
 **then**
19:          
$fid\;\leftarrow best_{\mathrm{match}};\;\mathrm{remove}\;\mathrm{from}\;L\;$
20:      
**else**
21:          
i←i+1, fid←i
22:          Initialize new EKF tracker23:      
**end if**
24:      
$M\left[{id}_{j}\right]\leftarrow fid$
25:      
$T\left[fid\right]\leftarrow EKF\;tracker$
26:      
**else**
27:          
$fid\leftarrow M\left[{id}_{j}\right]$
28:      
**end if**
29:      
**EKF Prediction:**

           ${\hat{x}}_{k}^{-}=f\left({\hat{x}}_{k-1}\right),\;\;P_{k}^{-}=F_{k}P_{k-1}F_{k}^{T}+Q_{k}$
30:      
**EKF Update:**

           $K_{k}=P_{k}^{-}H_{k}^{T}{\left(H_{k}P_{k}^{-}H_{k}^{T}+R_{k}\right)}^{-1}$
           ${{\hat{x}}_{k}=\hat{x}}_{k}^{-}+K_{k}\left(z_{j}-h\left({\hat{x}}_{k}^{-}\right)\right),\;\;P_{k}=\left(I-K_{k}H_{k}\right)P_{k}^{-}$
31:      
**If** 
$\left\|{\left[v\cos\theta ,v\sin\theta \right]}^{T}\right\|>10^{-4}$
 **then**
32:          
$\theta \leftarrow \;\tan^{-1}2(v\sin\theta ,v\cos\theta )$
33:      
**end if**
34:      Save ${\hat{x}}_{k}$ for output35:    
**end for**
36:     **for** each fid∉T not seen this frame **do**37:      
**If** 
fid∉L
 **then**
38:          
$L\left[fid\right]\;\leftarrow ({EKF}_{k},k)$
39:      
**else if** 
$k-L\left[fid\right].last\text{\_}seen>L$
 **then**
40:          Remove tracker from T and L
41:      
**end if**
42:    
**end for**
43: **end while**

### 2.5. ByteTrack Tracking Architecture

ByteTrack is a recent multi-object tracking algorithm that extends the SORT framework by incorporating both high-confidence and low-confidence detections into its data association process, significantly improving recall and robustness in dense and occluded environments [59,60]. Operating in a tracking-by-detection paradigm, detections {bi} with confidence scores {si} are received from an object detector such as YOLO11. These are divided into two groups: high-confidence detections with scores $s_{i}>\tau$, which are used in primary matching, and low-confidence detections where $\eta <s_{i}\leq \tau$, which serve in a secondary matching stage to recover missed objects and reduce false negatives [60].

The state of each tracked object is represented by a 6-dimensional vector:

(22) $$x={\left[x_{c},y_{c},w,h,{\dot{x}}_{c},{\dot{y}}_{c}\right]}^{T}$$

where $\left(x_{c},y_{c}\right)$ are the bounding box centre coordinates, w  and h represent the bounding box width and height, and the remaining terms represent their respective velocities [61]. The motion prediction follows a Kalman Filter model with constant velocity assumptions, and the process noise covariance accounts for the uncertainties in object motion dynamics [61]. Motion prediction is performed using a Kalman Filter under a constant velocity assumption:

(23) $${\hat{x}}_{k|k-1}={Fx}_{k-1}$$

(24) $$P_{k|k-1}=FP_{k-1}F^{T}+Q$$

where F is the state transition matrix and Q is the process noise covariance.

#### 2.5.1. ByteTrack Detection-to-Track Association

Association is performed in two sequential stages using the Hungarian algorithm. In the first stage, high-confidence detections are matched to active tracks by minimizing Intersection-over-Union (IoU) distance:

(25) $$d_{IoU}^{\left(i,j\right)}=1-IoU(b_{i},b_{j})$$

Matches with IoU below a predefined threshold are discarded. In the second stage, unmatched tracks from stage one are matched with low-confidence detections using IoU, enabling the recovery of objects missed in the first stage.

#### 2.5.2. Kalman Filter Update

For matched tracks, the Kalman Filter corrects predictions with the incoming detections:

(26) $$y_{k}=z_{k}-H{\hat{x}}_{k|k-1}$$

(27) $$K_{k}=P_{k|k-1}H^{T}\left(HP_{k|k-1}H^{T}+R\right)-1$$

(28) $${\hat{x}}_{k|k}={\hat{x}}_{k|k-1}+K_{k}y_{k}$$

(29) $$P_{k|k}=(I-K_{k}H)P_{k|k-1}$$

where H is the observation matrix, $K_{k}$ the Kalman gain, and R the measurement noise covariance matrix.

**Figure 3:**
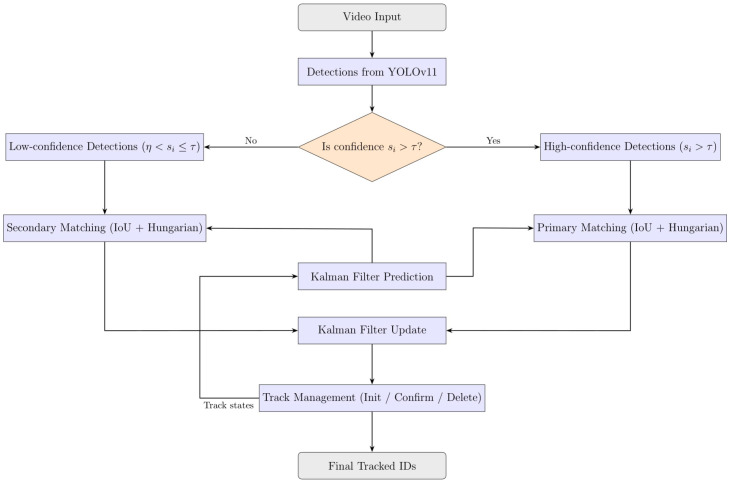
Overview of the ByteTrack algorithm.

Tracks are initiated from unmatched high-confidence detections and are confirmed after multiple consecutive matches to reduce false positives. Tracks that remain unmatched beyond a fixed number of frames are deleted. This conservative lifecycle management effectively reduces identity switches and enhances tracking stability. Figure 3 shows an overview of the ByteTrack algorithm.

### 2.6. DeepSORT Tracking Architecture

**Figure 4:**
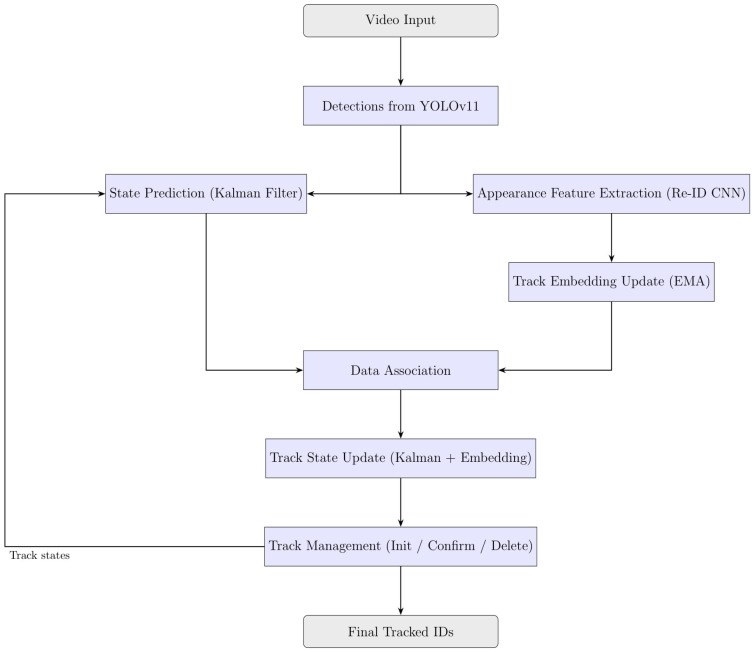
Overview of the DeepSORT algorithm.

DeepSORT extends the SORT framework by integrating appearance-based Re-Identification (Re-ID) features into the association process, improving identity preservation in scenarios where motion cues alone are insufficient, such as in dense or occluded sperm tracking environments [29,62]. Figure 4 illustrates an overview of the DeepSORT algorithm.

#### 2.6.1. DeepSORT Input and Detection Filtering

Like other tracking-by-detection methods, DeepSORT takes detections from YOLO11, each consisting of a bounding box and confidence score. For each detection, a 128-dimensional appearance embedding vector $f_{i}$ is extracted using a CNN-based Re-ID model [62,63]. These embeddings encode visual characteristics to help distinguish between similar objects when spatial overlap or motion ambiguity occurs.

#### 2.6.2. DeepSORT State Representation and Prediction

Each tracked object is modelled using an 8-dimensional state vector similar to ByteTrack and SORT, consisting of the bounding box centre coordinates $\left(x_{c},y_{c}\right)$, width w, height h, and their corresponding velocities $\left({\dot{x}}_{c},{\dot{y}}_{c},\dot{w},\dot{h}\right)$. The state evolves over time following a constant velocity model, with predictions generated by a Kalman Filter that accounts for process noise and measurement uncertainty [49,61]. This motion model enables robust position estimation even during temporary occlusions or missed detections.

#### 2.6.3. Appearance Feature Extraction

To maintain stable appearance information, DeepSORT updates the track’s appearance embedding using an Exponentially Weighted Moving Average (EMA):

(30) $$e_{k}=\alpha e_{k-1}+(1-\alpha )f_{k}$$

where α∈[0.9, 0.95] balances recent and historical appearance data [62]. This smooths out abrupt visual changes and reduces noise in the appearance model.

#### 2.6.4. DeepSORT Detection-to-Track Association

Data association uses a combination of motion and appearance similarity. Motion similarity is measured using the Mahalanobis distance between predicted and observed states, while appearance similarity is measured by cosine distance:

(31) $$d_{cos}(e_{i},f_{j})=1-\frac{e_{i}\cdot f_{j}}{∥e_{i}∥∥f_{j}∥}$$

These metrics are combined into a unified cost matrix. The Hungarian algorithm then finds the optimal matching that minimizes the total cost, effectively balancing geometric proximity and appearance similarity [29,62]. Tracks are confirmed only after multiple consecutive matches to reduce false positives, and lost tracks are removed after exceeding a set age threshold. The inclusion of appearance features significantly improves re-identification after occlusions, reducing ID switches and improving long-term tracking continuity in dense sperm videos [62,63].

## 3. Results and Discussion

We evaluated the proposed EKF-BoT-SORT tracker against BoT-SORT, ByteTrack, and DeepSORT using the VISEM dataset. This dataset contains 85 videos of live sperm. To obtain optimal performance and stability in BoT-SORT across different densities, we optimized the configuration of high and low thresholds to *τ* = 0.50, *η* = 0.10. This configuration showed the best overall balance between recall and track continuity and was used for all metrics. The evaluation included qualitative inspection of tracking sequences and quantitative analysis using standard Multi-Object Tracking (MOT) metrics. The focus was on identity preservation, track continuity, real-time performance, and detection accuracy.

**Figure 5:**
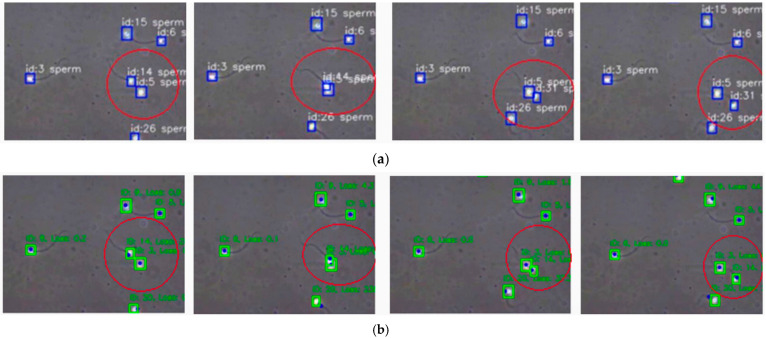
Example of ID switch correction. (**a**) BoT-SORT, the circled sperm changes from ID 14 to ID 31 after occlusion. (**b**) EKF-BoT-SORT preserves the original ID 14, maintaining trajectory continuity.

A direct visual comparison highlights the benefit of integrating EKF. Figure 5 shows the same video segment tracked with BoT-SORT (top) and EKF-BoT-SORT (bottom). In the baseline, the highlighted sperm changes its ID from 14 to 31 after a brief occlusion. With EKF verification, the original ID is retained, producing an uninterrupted trajectory. By predicting the sperm’s motion during the dropout and reassigning the correct ID upon re-detection, EKF eliminates the fragmentation and overcounting common in baseline trackers.

### 3.1. Identity Preservation

**Figure 6:**
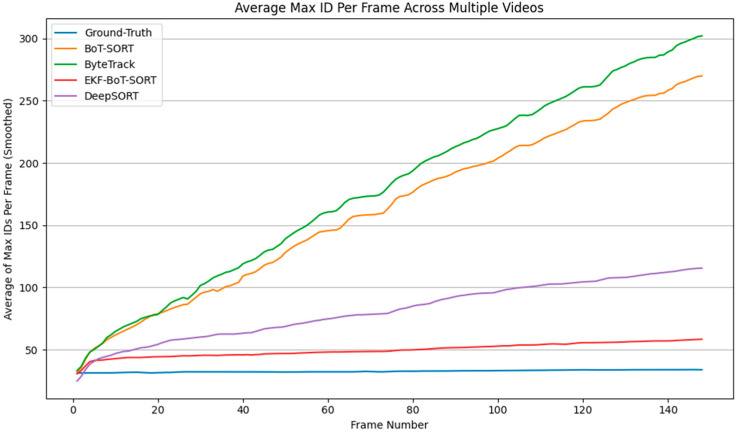
Tracking comparison shows unique sperm IDs detected in each frame.

Figure 6 illustrates the cumulative number of unique IDs detected over time by each tracker. The ground-truth dataset reports an average of 34 unique sperm IDs across the sequence. BoT-SORT and ByteTrack significantly overcount, reaching 270 and 302 IDs, respectively. On the other hand, DeepSORT shows a much better performance compared to BoT-SORT and ByteTrack in terms of ID count, with an average of 115 IDs. This overcount is primarily due to re-identification failures.

In contrast, EKF-BoT-SORT demonstrates improved identity consistency, reporting only 58 IDs. Although this is still above ground truth, across all trackers, EKF-BoT-SORT achieved the lowest unique-ID inflation, reducing overcount from 7.94× the ground truth in BoT-SORT, 8.88× in ByteTrack, and 3.38× in DeepSORT to just 1.71×. In absolute terms, this corresponds to reducing extra IDs from 236 (BoT-SORT), 268 (ByteTrack), and 115 (DeepSORT) to only 34, reflecting a substantial improvement in identity continuity without compromising detection quality.

### 3.2. Track Duration Stability

**Figure 7:**
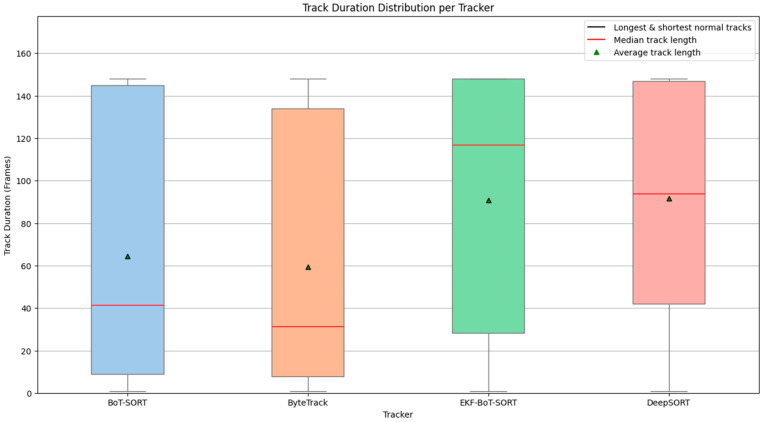
Track duration distribution per tracker.

Figure 7 presents the distribution of track durations (measured in frames) for four different tracking algorithms: BoT-SORT, ByteTrack, DeepSORT, and the proposed EKF-BoT-SORT. Each box shows the interquartile range (IQR) of track lengths, with the lower and upper edges representing the 25th and 75th percentiles. The red horizontal line marks the median track duration, while the green triangle shows the mean. Whiskers extend to the shortest and longest non-outlier tracks, giving an idea of how variable each tracker is in keeping identities consistent.

BoT-SORT and ByteTrack both have low median values, which means most of their tracks are short. This comes from frequent ID switches, early terminations, or not being able to recover a track after a missed detection or occlusion. Their whiskers show they can sometimes produce long tracks, but these are far less common than short, broken ones.

DeepSORT manages some of the longest tracks in the dataset, which is why its upper whisker is noticeably higher. However, its median is still lower than EKF-BoT-SORT, so those long tracks are exceptions rather than the usual case.

EKF-BoT-SORT stands out with a higher median and a tighter IQR shifted toward longer tracks. This means most of its tracks last longer before being broken. On average, EKF-BoT-SORT reaches 91.3 frames per track, which is 23% higher than BoT-SORT and 32% higher than ByteTrack. DeepSORT’s mean is slightly higher at 99.2 frames (≈8.6% more than EKF-BoT-SORT), but its higher variability and lower median show it does not maintain this performance consistently.

For sperm motility analysis, this consistency matters more than a few extra-long tracks, because stable, unbroken trajectories give more reliable and meaningful motion data for downstream calculations.

### 3.3. Multi-Object Tracking (MOT) Metrics

Table 3 compares the Multi-Object Tracking (MOT) metrics for all four trackers. EKF-BoT-SORT achieves the highest scores in IDF1 (84.84%), ID Precision (84.71%), and ID Recall (86.18%), outperforming BoT-SORT by 4.54%, 6%, and 4.46%, respectively. This means it is better at both assigning the correct ID and keeping it consistent over time.

ID switches drop from 176 in BoT-SORT, 178 in ByteTrack, and 133 in DeepSORT to 132 with EKF-BoT-SORT, showing that the EKF layer is effective at correcting re-identification errors caused by occlusions or missed detections. Compared to the baseline trackers, EKF-BoT-SORT not only produces fewer ID switches but also maintains higher identity-related metrics across the board.

Precision and recall stay high across all trackers, with EKF-BoT-SORT (87.95% precision, 90.77% recall) performing close to BoT-SORT and ByteTrack and better than DeepSORT. This suggests that adding EKF does not adversely affect detection quality. MOTA remains close to the baseline (41.80% vs. 42.47% for BoT-SORT), with the small drop likely due to a conservative reassignment strategy that favours avoiding false ID matches over chasing every detection. MOTP is essentially unchanged (~0.47), meaning localization accuracy stays the same.

**Figure 8:**
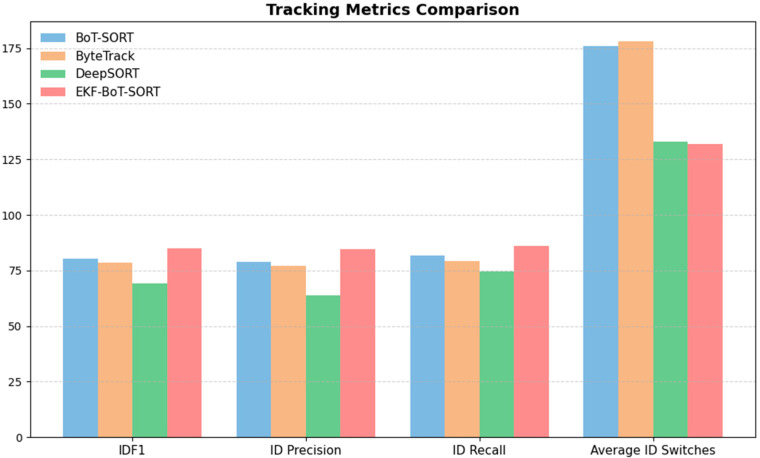
Tracking metrics comparison. EKF-BoT-SORT achieves the highest IDF1, ID Precision, and ID Recall, while reducing ID switches compared to all baselines.

The metrics show that EKF-BoT-SORT keeps the strong detection performance of BoT-SORT while delivering more stable identities and fewer switches, which is critical for downstream sperm motility analysis. Figure 8 provides a visual comparison of the key identity-related metrics (IDF1, ID Precision, ID Recall, and average ID switches) across all four trackers.

The ID overcount and average track duration for all trackers are summarized in Table 4. EKF-BoT-SORT achieves the lowest ID inflation, with only 1.71× the ground truth count (24 extra IDs), compared to 7.94× (236) for BoT-SORT, 8.88× (268) for ByteTrack, and 3.38× (81) for DeepSORT. This reduction in overcount reflects a substantial improvement in identity preservation. In terms of track duration, EKF-BoT-SORT produces an average of 91.3 frames per track, which is 23% longer than BoT-SORT and 32% longer than ByteTrack, while remaining competitive with DeepSORT’s 99.2-frame average.

**Figure 9:**
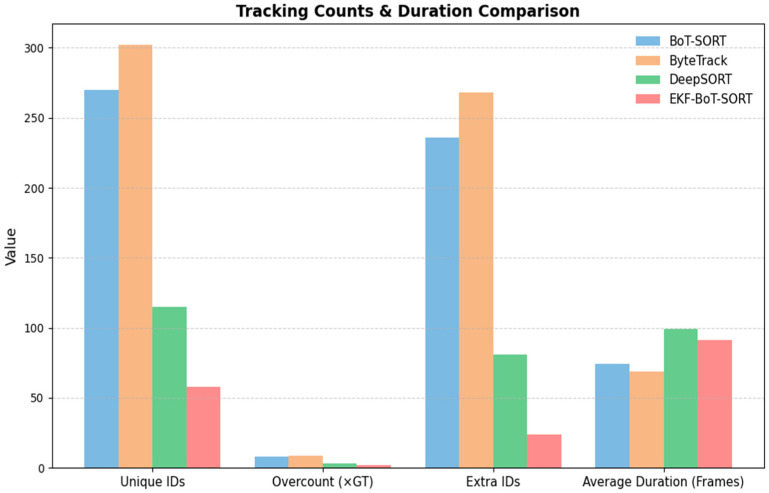
Tracking count and duration comparison of BoT-SORT, ByteTrack, DeepSORT, and EKF-BoT-SORT.

Similarly to Table 4, a clear illustration of this comparison is provided in Figure 9. The combination of reduced overcount and extended track continuity results in smoother, more biologically meaningful sperm trajectories, which are essential for accurate motility analysis.

### 3.4. Ablation Study on Motion State

The contribution of the heading-angle modelling in the motion state was evaluated by conducting a comparison of two different EKF state vector models under similar detection and association settings:

- a simplified velocity-only EKF with state vector

(32) [x,y,v]

- our proposed EKF with heading-angle

(33) [x,y,v,θ]

The results in Table 5 show that including the heading-angle improved the identity consistency in the tracks, increasing IDF1, ID precision, and ID recall, and reducing the number of ID switches from 200 to 132, showing the orientation component strengthens identity continuity. Detection metrics such as precision and recall remained unchanged.

By improving the short-term motion prediction, the heading-angle modelling shows improvement in the tracker performance and helps the trajectory continuity.

### 3.5. Real-Time Performance

Hardware Setup: All experiments were conducted on a system equipped with an Intel^®^ Core™ i9-14900HX (24 cores, up to 5.8 GHz) (Intel Corporation, Santa Clara, CA, USA), 32 GB DDR5 RAM (4800 MHz) (Corsair Components, Fremont, CA, USA), and an NVIDIA^®^ GeForce RTX™ 4070 GPU with 8 GB GDDR6 VRAM (NVIDIA Corporation, Santa Clara, CA, USA). Storage was provided by a 2 TB Samsung 990 PRO NVMe SSD (Samsung Electronics Co., Ltd., Suwon, South Korea). The operating system was Windows 10 Professional 64-bit.

We evaluated the runtime efficiency of each tracker to assess their suitability for real-time sperm analysis; Table 6 shows a detailed comparison of all trackers. Average throughput (frames per second, FPS) was calculated across all test videos and compared against the recorded video frame rates (~48–50 FPS). BoT-SORT maintained a mean processing speed of 59.8 FPS, consistently exceeding the video playback rate and thus operating in real time. EKF-BoT-SORT achieved a slightly higher average of 62.4 FPS, likely due to reduced data-association complexity when ID continuity is preserved. This indicates that the EKF layer not only avoids adding computational overhead but can also marginally improve runtime efficiency while enhancing tracking robustness. ByteTrack was the fastest overall, averaging 95.2 FPS, but this came at the cost of higher ID overcount and reduced trajectory continuity. DeepSORT showed the slowest performance compared to others, mainly due to appearance feature extraction and the extra data association steps, resulting in an FPS of 10.

The framework achieves real-time performance on a high-end workstation, but on lower-resource clinical or laboratory conditions, it is possible to achieve this. The framework is computationally efficient because it is a detection-based tracking system, and the overall real-time performance is mainly dependent on the YOLO detector used. Replacing the current YOLO model with more compact variants (e.g., YOLO11n or YOLO11s) allows the pipeline to run efficiently on mid-range CPUs or embedded GPUs such as NVIDIA Jetson devices while maintaining acceptable accuracy [24].

In terms of scalability, the EKF-BoT-SORT updates each detection independently, which results in the computational complexity being linear, depending on the number of tracked objects. These characteristics make the real-time performance of the framework at higher sperm densities more stable and predictable.

**Figure 10:**
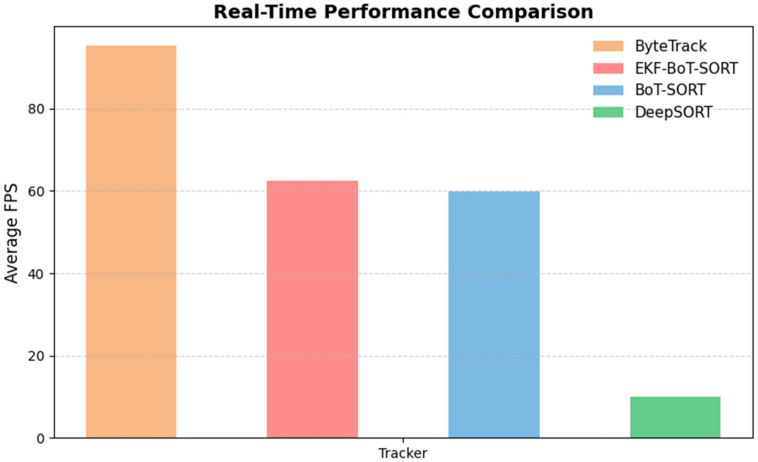
Real-Time performance comparison between BoT-SORT, ByteTrack, DeepSORT, and EKF-BoT-SORT.

Overall, EKF-BoT-SORT offers a balanced trade-off between accuracy and speed, achieving measurable gains in identity preservation while maintaining the ability to process videos near or above acquisition rates—an essential requirement for automated motility assessment in clinical and research workflows. Figure 10 provides a visual presentation of the real-time performance of each tracker compared to ByteTrack, showing the highest performance, and DeepSORT showing the slowest performance out of all the trackers. EKF-BoT-SORT shows a balanced trade-off between speed and accuracy.

### 3.6. Evaluation of Derived Motility Parameters

For further evaluation of the clinical relevance of the proposed framework and the biological interpretability of the resulting trajectories, standard motility parameters were computed for each sperm from the tracking output of BoT-SORT and the proposed EKF-BoT-SORT framework, and compared to the manually verified ground-truth trajectories.

The calculated parameters include curvilinear velocity (VCL), straight-line velocity (VSL), and linearity (LIN), which are common in CASA systems. These metrics were calculated using the following definition [64]:

(34) $$\mathrm{VCL}=\frac{\sum i\sqrt{(x_{i+1}-x_{i}{)}^{2}+(y_{i+1}-y_{i}{)}^{2}}}{T}$$

(35) $$\mathrm{VSL}=\frac{\sqrt{(x_{\mathrm{end}}-x_{\mathrm{start}}{)}^{2}+(y_{\mathrm{end}}-y_{\mathrm{start}}{)}^{2}}}{T}$$

(36) $$\mathrm{LIN}=\frac{\mathrm{VSL}}{\mathrm{VCL}}$$

where x and y are showing the starting and end positions of sperm in that trajectory, and T is the trajectory duration in seconds. These parameters were identically calculated, and the average of the results is shown on Table 7.

The values of velocity calculated from the tracked trajectories were lower than those calculated from the ground truth. This difference is expected, and it is because the ground-truth tracks capture full oscillations in sperm motion, while the trackers are limited by the Yolo detection outputs and filter out some small-scale positional variation. The motility parameters calculated from the EKF-BoT-SORT trajectory outputs are closer to the ground truth than the parameters calculated from BoT-SORT trajectory outputs, which reflect improved trajectory continuity.

It is important to note that the EKF-BoT-SORT tracker combines each detection with motion prediction. This allows smooth frame-to-frame jitters and keeps the same correct sperm ID over time, which reduces noise in position measurement while keeping the real head movement of the sperm. As a result of this, the calculated VCL values are lower than the ground truth values, but the trajectories are more stable and realistic, which makes them more reliable for motility classification and further downstream analysis, supporting the potential application of the proposed framework in clinical use. Because the centre point of the bounding boxes does not track the small oscillations of the sperm head, the tracking-based velocity estimates will be lower compared to the ground truth.

### 3.7. Performance Under Varying Frame Rates and Image Conditions

To investigate the performance of the proposed EKF-BoT-SORT tracker under different video input conditions, additional experiments were conducted under three controlled variants:

- Temporal downsampling to 12.5 fps (keep-every-4 frames),
- Temporal downsampling to 25 fps (keep-every-2 frames), and
- Visual degradation using Gaussian blur σ = 0.8.

In each of these scenarios, the tracker was evaluated across low-, medium-, and high-density videos, while varying the process and measurement noise scales (Q-scale  α, R-scale β) within {0.5, 1.0, 2.0}. The YOLO and base tracker configurations were maintained the same across all tests; the ground-truth annotations were downsampled to ensure temporal alignment with the videos. The results are presented in Appendix B (Table A4, Table A5 and Table A6).

With 12.5 fps, the performance of the tracker is significantly challenged by performing under reduced temporal resolution. In this setting, both detection continuity and ID continuity are tested, and the results in Appendix B, Table A4 show that performance remains stable under all variations in Q- and R-scale combinations. For low-density videos, the tracker maintains a strong performance with very few ID switches, which shows that in scenes where the objects are scattered, the tracker can maintain their identities effectively. In medium-density videos, the reduced temporal sampling led to a decrease in IDF1 and MOTA, which is mainly caused by fragmented associations in shorter visible trajectories. In high-density videos, the tracker achieved IDF1 ≈ 51% and MOTA ≈ 14%, which shows the difficulty in maintaining identity in more crowded scenes with frequent overlap and occlusion.

At 25 fps, the temporal resolution is doubled compared to the 12.5 fps test, which allows smoother motion continuity and more reliable association updates. The results in Appendix B, Table A5, show a clear improvement in both IDF1 and MOTA across all density levels, which confirms that higher frame rates help preserve identity consistency in sperm motion tracking. For low-density videos, the results show a near-optimal tracking stability with an average of IDF1 ≈ 80% and MOTA ≈ 63%, and only four identity switches. In medium-density videos, the increase in overlaps and occlusions causes a reduction in the overall IDF1 to ≈50% and MOTA to ≈17%. In high-density videos, the tracker maintained IDF1 ≈ 60% and MOTA ≈ 26%, showing that the EKF prediction mechanism can effectively perform in highly crowded scenes. In comparison with the 12.5 fps setting, the results confirm that the higher temporal resolution enhances the tracking continuity.

Under Gaussian blur degradation (σ = 0.8), the visual clarity of the videos is reduced, which introduces uncertainty in the detection bounding boxes and confidence. The results in Appendix B, Table A6, show that the EKF-BoT-SORT tracker remains stable. In low-density videos, the tracker achieved IDF1 ≈ 71% and MOTA ≈ 63%, which nearly matches the 25-fps performance. The small number of identity switches (≈5) suggests that blurred but isolated sperm can still be effectively associated across frames. In medium density scenes, the performance decreased to IDF1 ≈ 50% and MOTA ≈ 13%, showing the effect of combining visual blur and an increase in overlap and occlusion due to the higher sperm density. In high-density videos, the tracker performed with IDF1 ≈ 55% and MOTA ≈ 16%, indicating that even under dense and visually degraded conditions, the EKF prediction effectively sustains consistent identity tracking despite higher uncertainty.

Overall, the performance of the model remained stable across all densities with different variations in Q- and R-scale combinations, while the temporal downsampling and the clarity of the input videos can affect the model’s performance.

### 3.8. Failure Cases and Limitations

**Figure 11:**
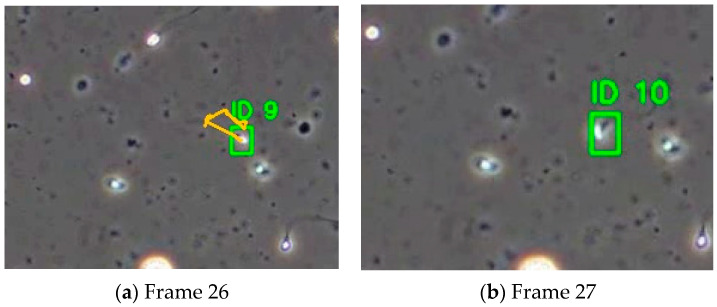
Failure example of EKF-BoT-SORT due to sudden motion changes.

Although EKF-BoT-SORT shows improvement in identity preservation and track continuity, there are still limitations to the performance of this framework. Figure 11 shows a failure scene where this framework cannot successfully maintain correct identities across frames due to long detection loss and a sudden change in sperm motion.

- High-density samples: At high densities exceeding 60–70 sperm per 640 × 480 frame, detection losses become longer and more frequent, along with more overlaps, causing longer occlusion. The performance of this method becomes less reliable; this may cause the detection prediction to be aligned with multiple candidates, causing identity swaps or dropped tracks.
- Sudden motion changes: The framework assumes locally constant velocity and heading direction. When a sperm suddenly changes direction or has a large acceleration, the distance between the new position and the predicted state goes above the threshold; therefore, the identity reassignment fails to reassign the correct ID to the sperm. In these situations, tracking performance relies on the detection model.
- Long detection losses: If a sperm is missing for too many frames, the uncertainty in the predicted position increases, and this causes the reliability in reassignment to decrease, resulting in the system terminating the track in order to prevent false re-identification.

These examples highlight the challenges still existing in maintaining identity continuity for objects with complex motion and occlusion, and motivations for future work.

The interpretability of EKF–BoT-SORT is different from feature-level explainability that is more commonly used in deep learning models such as Grad-CAM or Shapley-value–based attribution, but it achieves interoperability through physical motion modelling, providing visual insights into how the tracker reasons about sperm movement. This transparency allows explainability through data; by providing clear trajectories, the motility parameters (VCL, VSL, LIN) calculated from the EKF-BoT-SORT outputs are biologically meaningful and explainable.

The proposed EKF-assisted identity reassignment framework also has potential beyond sperm tracking. It addresses a core challenge in microscopic imaging: maintaining consistent object identities under nonlinear motion and frequent occlusions in low-contrast environments. Because of its modular design and the model’s reliance on motion models, the framework can be integrated with various detection and tracking pipelines, extending its applicability to other domains where the motion behaviour has more influence in comparison with appearance-based cues. For example, in cell and bacteria tracking [65,66], objects move in irregular motion similar to sperm, the prediction-based tracking framework can reduce ID switches and broken paths. In embryo and oocyte monitoring in reproductive medicine [67], the method could help with maintaining a consistent identity across a long time-lapse sequence to perform cell monitoring. In microorganism motility analysis in behavioural studies, such as algae or protozoa [68], which often swim in nonlinear and curved patterns, the EKF-BoT_SORT model can potentially provide the movement trajectories more accurately.

Beyond biological imaging, the same approach can support micro-robot swarm tracking and particle motion analysis in experimental fluid dynamics, where accurate trajectory continuity is essential [69]. By focusing on prediction-based identity management rather than appearance cues, EKF–BoT-SORT offers a general solution for multi-object tracking tasks where motion dynamics drive re-identification accuracy. Because the framework works independently of detector or training data, it can be combined with different imaging setups and tracking pipelines without extra retraining. This makes EKF-BoT-SORT a flexible framework and general tool for studying nonlinear motion.

## 4. Conclusions

The aim of this study was to improve the accuracy and reliability of sperm tracking for automated fertility assessment by addressing identity preservation challenges common in conventional tracking systems. To achieve this, we developed a robust two-layer tracking framework that enhances BoT-SORT with an Extended Kalman Filter (EKF), specifically designed to model nonlinear sperm dynamics and verify identities over time. This approach significantly reduces identity fragmentation, overcounting, and tracking noise in challenging microscopy conditions.

Experimental evaluation on the VISEM dataset showed that the proposed EKF-BoT-SORT framework improves IDF1 from 80.30% to 84.84% (+4.54%), increases average track duration from 74.4 to 91.3 frames (+23%), and reduces ID switches from 176 to 132 (–25%) compared to baseline BoT-SORT. Compared to ByteTrack and DeepSORT, our method consistently achieved the lowest unique-ID inflation and maintained longer, more stable trajectories without sacrificing detection precision or recall.

These results suggest that the method can maintain long-term identity consistency and may enable more biologically meaningful trajectories, which is critical for accurate sperm motility assessment. Future work will focus on incorporating richer motion cues, testing on denser and more diverse datasets, and deploying the system in real-time clinical environments.

## Figures and Tables

**Table 1:** Qualitative comparison of tracking performance across different tracker types, showing their key mechanisms, real-time performance, and overall practical suitability.

Base Method	Tracker Type	Category	Key Mechanism	Real-Time Performance	Practical Suitability
Kalman Filter	BoT-SORT [28]	Decoupled: Tracking by Detection (TBD)	Two-stage association with Re-ID features and camera-motion compensation	Yes	Real-time and efficient, but still prone to ID switches under rapid or nonlinear motion
Kalman Filter	ByteTrack [27,34]	Decoupled: Tracking by Detection (TBD)	Two-stage IoU association using high- and low-score detections to reduce false negatives	Yes	Fast and accurate in simple motion, but unstable under dense occlusion or abrupt motion
Kalman filter + deep appearance	DeepSORT [29]	Decoupled: Tracking by Detection (TBD)	Re-ID association via appearance embedding	No	Robust appearance modelling but computationally heavy and not real-time
Transformer-based	TransTrack [30,34]	End-to-End (E2E)	Track-query and image-feature cross-attention for joint detection and tracking	No	High accuracy but below real-time; heavy transformer inference
Graph Transformer	TransMOT [31]	End-to-End (E2E)	Spatio-temporal self-attention on object feature graphs	No	Context-aware but computationally heavy; not real-time
Transformer (DETR-style)	MOTR [33,34]	End-to-End (E2E)	Recurrent track-query propagation across video frames	No	Heavy computation; unsuitable for real-time microscopy
Transformer + deformable attention	HDE-Track [34]	End-to-End (E2E)	Spatio-temporal deformable attention across multi-scale features	No	Accurate but slow and resource-intensive; impractical for live tracking

**Table 2:** Sensitivity of EKF ID reassignment threshold (*τ*) across densities.

Video Density	*τ*	IDF1	IDSW
Low	15	86.0479	8
	30	88.6848	7
	45	88.6848	7
Medium	15	53.0763	27
	30	55.3039	15
	45	55.3039	15
High	15	60.6828	43
	30	61.6965	32
	45	61.6812	31

**Table 3:** Quantitative comparison of tracking performance metrics across different trackers, showing that EKF-BoT-SORT achieves the highest IDF1, ID precision, and ID recall with the fewest ID switches, indicating superior identity preservation and overall tracking robustness.

Metric	BoT-SORT	ByteTrack	DeepSORT	EKF-BoT-SORT
IDF1	80.2995%	78.3883%	69.1045%	84.8408%
ID Precision	78.7111%	77.2144%	63.8659%	84.7115%
ID Recall	81.7137%	79.2776%	74.68%	86.1778%
Precision	88.4339%	87.5924%	70.806%	87.9534%
Recall	91.443%	89.4988%	82.3119%	90.7709%
Average ID Switches	176	178	133	132
MOTA	42.4722%	40.2926%	38.5905%	41.8034%
MOTP	0.467837	0.470183	0.483309	0.46695

**Table 4:** Comparison of ID overcount and average track duration across trackers, showing that EKF-BoT-SORT achieves the lowest overcount with high duration, indicating effective identity preservation without excessive fragmentation.

Tracker	Unique IDs	Overcount (×GT)	Extra IDs	Average Duration
BoT-SORT	270	7.94×	+236	74.4
ByteTrack	302	8.88×	+268	68.9
DeepSORT	115	3.38×	+81	99.2
EKF-BoT-SORT	58	1.71×	+24	91.3

**Table 5:** Ablation study on the effect of heading-angle on model performance.

State Vector	IDF1	ID Precision	ID Recall	Average ID Switches
Original	84.8408%	84.7115%	86.1778%	132
No Heading	80.20%	84.20%	76.10%	200

**Table 6:** Average runtime performance (frames per second, FPS) of each tracker across all test videos. Recorded video frame rates were ~48–50 FPS. EKF-BoT-SORT maintains real-time capability with minimal overhead compared to BoT-SORT.

Tracker	Avg FPS	Relative to Video FPS	Real-Time Capable
ByteTrack	95.2	1.92×	Yes
EKF-BoT-SORT	62.4	1.26×	Yes
BoT-SORT	59.8	1.22×	Yes
DeepSORT	10	0.14×	No

**Table 7:** Motility parameters calculated from BoT-SORT, EKF-BoT-SORT, and ground truth.

Tracker	VCL (px/s)	VSL (px/s)	LIN
BoT-SORT	357.7572	122.5268	2.919827
EKF-BoT-SORT	83.82887	30.57383	2.74185
Ground Truth	110.8941615	24.9363	4.447098

## Data Availability

The data presented in this study are openly available in [VISEM] [https://doi.org/10.5281/zenodo.2640506], reference number [70].

## Appendix A

**Table A1:** Sensitivity analysis of high-confidence threshold (*τ*) on tracking performance. Tracking performance is reported on the VISEM dataset for varying values of *τ*, with low-confidence threshold *η* fixed at 0.10.

Video Density	*η*	*τ*	IDF1	IDSW	MOTA	MOTP
Low	0.10	0.25	83.3831	9	80.0621	0.316043
	0.10	0.50	83.5555	9	80.4609	0.315847
	0.10	0.60	88.7420	9	81.0169	0.31602
Medium	0.10	0.25	44.8121	90	−2.8685	0.371946
	0.10	0.50	44.7857	34	1.3611	0.376077
	0.10	0.60	40.6163	29	1.4209	0.379643
High	0.10	0.25	55.3717	154	15.7022	0.361436
	0.10	0.50	54.7822	51	20.4950	0.366644
	0.10	0.60	51.2485	41	18.3951	0.368752

**Table A2:** Sensitivity analysis of low-confidence threshold (*η*) on tracking performance. Performance shown with high-confidence threshold *τ* fixed at 0.50.

Video Density	*η*	*τ*	IDF1	IDSW	MOTA	MOTP
Low	0.05	0.50	83.5555	9	80.4609	0.315847
	0.10	0.50	83.5555	9	80.4609	0.315847
	0.20	0.50	83.7503	9	80.9098	0.315847
Medium	0.05	0.50	44.7857	34	1.3611	0.376077
	0.10	0.50	44.7876	34	1.3611	0.376077
	0.20	0.50	45.0270	31	3.9157	0.375722
High	0.05	0.50	54.7822	51	20.4950	0.366644
	0.10	0.50	54.7822	51	20.4950	0.366644
	0.20	0.50	55.5793	50	21.4893	0.365799

## Appendix B

Table A3 shows the full sensitivity analysis conducted on the process noise covariance Q and measurement noise covariance R. Both parameters were varied across scaling factors of α,β∈{0.5, 1.0, 2.0} while all other tracker settings remained fixed. This evaluation was performed across low-, medium-, and high-density sperm videos.

**Table A3:** Sensitivity analysis conducted on the process noise covariance Q  and measurement noise covariance R.

Video Density	Q-Scale (α)	R-Scale (β)	IDF1	IDSW	MOTA	MOTP
Low	0.5	0.5	88.6848	7	80.1528	0.323142
	0.5	1.0	88.6848	7	80.1528	0.323142
	0.5	2.0	88.6438	7	80.1116	0.323142
	1.0	0.5	88.6848	7	80.1528	0.323142
	1.0	1.0	88.6848	7	80.1528	0.323142
	1.0	2.0	88.6438	7	80.1116	0.323142
	2.0	0.5	88.6848	7	80.1528	0.323142
	2.0	1.0	88.6848	7	80.1528	0.323142
	2.0	2.0	88.6438	7	80.1116	0.323142
Medium	0.5	0.5	55.3150	15	17.7708	0.369987
	0.5	1.0	55.3039	15	17.7602	0.369987
	0.5	2.0	55.0496	17	17.7284	0.369987
	1.0	0.5	55.3150	15	17.7708	0.369987
	1.0	1.0	55.3039	15	17.7602	0.369987
	1.0	2.0	55.2376	16	17.7390	0.369987
	2.0	0.5	55.3150	15	17.7708	0.369987
	2.0	1.0	55.3039	15	17.7602	0.369987
	2.0	2.0	55.2376	16	17.7390	0.369987
High	0.5	0.5	61.6965	31	29.8298	0.359590
	0.5	1.0	61.7269	32	29.8227	0.359590
	0.5	2.0	61.5654	33	29.8045	0.359590
	1.0	0.5	61.6965	31	29.8298	0.359590
	1.0	1.0	61.6965	32	29.8227	0.359590
	1.0	2.0	61.5197	33	29.8045	0.359590
	2.0	0.5	61.5389	32	29.8237	0.359590
	2.0	1.0	61.6965	32	29.8227	0.359590
	2.0	2.0	61.6775	32	29.8166	0.359590

Table A4, Table A5 and Table A6 provide the full quantitative results for the experiments described in Section 3.7. These tables include performance under reduced frame rates (12.5 fps and 25 fps) and visual degradation using Gaussian blur.

**Table A4:** Performance of EKF–BoT-SORT under 12.5 fps downsampling.

Video Density	Q-Scale (α)	R-Scale (β)	IDF1	IDSW	MOTA	MOTP
Low	0.5	0.5	66.1033	5	42.6024	0.324718
	0.5	1.0	66.1033	5	42.6024	0.324718
	0.5	2.0	65.5832	7	42.2718	0.324718
	1.0	0.5	66.1033	5	42.6024	0.324718
	1.0	1.0	66.1033	5	42.6024	0.324718
	1.0	2.0	65.5832	7	42.2718	0.324718
	2.0	0.5	68.0101	3	42.933	0.324718
	2.0	1.0	66.1033	5	42.6024	0.324718
	2.0	2.0	64.2687	7	42.1214	0.324718
Medium	0.5	0.5	38.1207	11	−1.438	0.377121
	0.5	1.0	37.921	12	−1.564	0.377121
	0.5	2.0	38.1207	9	−1.311	0.377121
	1.0	0.5	38.0207	10	−1.396	0.377121
	1.0	1.0	38.0207	12	−1.523	0.377121
	1.0	2.0	38.021	10	−1.351	0.377121
	2.0	0.5	38.1207	8	−1.226	0.377121
	2.0	1.0	38.0207	11	−1.438	0.377121
	2.0	2.0	38.1706	9	−1.269	0.377121
High	0.5	0.5	51.4518	13	14.2237	0.378181
	0.5	1.0	51.3719	14	14.1993	0.378181
	0.5	2.0	51.5010	15	14.1141	0.378181
	1.0	0.5	51.4518	13	14.2237	0.378181
	1.0	1.0	51.4518	13	14.2237	0.378181
	1.0	2.0	51.5809	15	14.1386	0.378181
	2.0	0.5	51.3872	14	14.1954	0.378181
	2.0	1.0	51.4518	13	14.2237	0.378181
	2.0	2.0	51.5809	15	14.1386	0.378181

**Table A5:** Performance of EKF–BoT-SORT under 25 fps downsampling.

Video Density	Q-Scale (α)	R-Scale (β)	IDF1	IDSW	MOTA	MOTP
Low	0.5	0.5	79.8891	4	63.2051	0.32915
	0.5	1.0	79.8891	4	63.2051	0.32915
	0.5	2.0	79.8891	4	63.2051	0.32915
	1.0	0.5	79.8891	4	63.2051	0.32915
	1.0	1.0	79.8891	4	63.2051	0.32915
	1.0	2.0	79.8891	4	63.2051	0.32915
	2.0	0.5	79.8891	4	63.2051	0.32915
	2.0	1.0	79.8891	4	63.2051	0.32915
	2.0	2.0	79.8891	4	63.2051	0.32915
Medium	0.5	0.5	50.1189	13	17.4599	0.374471
	0.5	1.0	49.5374	12	17.4798	0.374471
	0.5	2.0	49.543	14	17.3987	0.374471
	1.0	0.5	50.4742	11	17.5646	0.374471
	1.0	1.0	49.9294	12	17.5022	0.374471
	1.0	2.0	49.543	14	17.3987	0.374471
	2.0	0.5	50.3609	10	17.607	0.374471
	2.0	1.0	49.8544	12	17.501	0.374471
	2.0	2.0	50.3043	12	17.501	0.374471
High	0.5	0.5	59.5507	16	26.4196	0.371247
	0.5	1.0	59.9684	14	26.4823	0.371247
	0.5	2.0	59.8591	15	26.4681	0.371247
	1.0	0.5	60.0153	14	26.4965	0.371247
	1.0	1.0	59.93	15	26.4459	0.371247
	1.0	2.0	59.5194	17	26.4054	0.371247
	2.0	0.5	59.8278	14	26.4823	0.371247
	2.0	1.0	59.7809	15	26.4681	0.371247
	2.0	2.0	59.6091	16	26.4397	0.371247

**Table A6:** Performance of EKF–BoT-SORT under Gaussian blur degradation.

Video Density	Q-Scale (α)	R-Scale (β)	IDF1	IDSW	MOTA	MOTP
Low	0.5	0.5	70.7424	5	62.8542	0.266271
	0.5	1.0	70.7424	5	62.8542	0.266271
	0.5	2.0	70.7424	5	62.8542	0.266271
	1.0	0.5	70.7424	5	62.8542	0.266271
	1.0	1.0	70.7424	5	62.8542	0.266271
	1.0	2.0	70.7424	5	62.8542	0.266271
	2.0	0.5	70.7424	5	62.8542	0.266271
	2.0	1.0	70.7424	5	62.8542	0.266271
	2.0	2.0	70.7424	5	62.8542	0.266271
Medium	0.5	0.5	49.6359	17	13.5899	0.377122
	0.5	1.0	49.8138	17	13.6021	0.377122
	0.5	2.0	49.5021	17	13.5899	0.377122
	1.0	0.5	49.6359	17	13.5899	0.377122
	1.0	1.0	49.8138	17	13.6021	0.377122
	1.0	2.0	49.3241	18	13.5777	0.377122
	2.0	0.5	49.6359	17	13.5899	0.377122
	2.0	1.0	49.8138	17	13.6021	0.377122
	2.0	2.0	49.4767	17	13.5899	0.377122
High	0.5	0.5	54.7849	14	16.1906	0.375561
	0.5	1.0	54.6209	15	16.1846	0.375561
	0.5	2.0	54.7849	14	16.1906	0.375561
	1.0	0.5	54.7849	14	16.1906	0.375561
	1.0	1.0	54.7849	14	16.1906	0.375561
	1.0	2.0	54.7849	14	16.1906	0.375561
	2.0	0.5	54.7849	14	16.1906	0.375561
	2.0	1.0	54.7849	14	16.1906	0.375561
	2.0	2.0	54.7849	14	16.1906	0.375561
